# Circulating T Cells Exhibit Different TIM3/Galectin-9 Expression in Patients with Obesity and Obesity-Related Diabetes

**DOI:** 10.1155/2020/2583257

**Published:** 2020-10-15

**Authors:** Lili Sun, Shengyi Zou, Sisi Ding, Xuan Du, Yu Shen, Cuiping Liu, Bimin Shi, Xueguang Zhang

**Affiliations:** ^1^Jiangsu Institute of Clinical Immunology & Jiangsu Key Laboratory of Clinical Immunology, First Affiliated Hospital of Soochow University, No. 708 Renmin Road, Suzhou, 215006 Jiangsu, China; ^2^Departments of Endocrinology, The First Affiliated Hospital of Soochow University, Suzhou, 215006 Jiangsu, China

## Abstract

**Aims:**

Obesity is highly associated with type 2 diabetes mellitus (T2DM). The TIM3/galectin-9 pathway plays an important role in immune tolerance. Herein, we aimed to investigate the expression of TIM3 and galectin-9 in peripheral blood and to evaluate their clinical significance in patients with obesity and obesity-related T2DM.

**Methods:**

We performed flow cytometry on peripheral blood samples from healthy donors (HC), patients with simple obesity (OB), and patients with obesity comorbid T2DM (OD). The expression of TIM3 on CD3^+^, CD4^+^, and CD8^+^ T cells was determined. The level of galectin-9 in plasma was detected by ELISA.

**Results:**

We demonstrated the enhancement of TIM3 on CD3^+^, CD4^+^, and CD8^+^ T cells in the OB group when compared with healthy controls, while it was decreased significantly in the OD group. The TIM3^+^CD8^+^ T cells of the OB group were positively correlated with risk factors including BMI, body fat rate, and hipline. The concentration of galectin-9 of the OD group in plasma was significantly higher than that of healthy donors and the OB group. Moreover, the level of galectin-9 of the OD group was positively correlated with fasting insulin and C-peptide, which were two clinical features that represented pancreatic islet function in T2DM.

**Conclusions:**

Our results suggested that TIM3 and galectin-9 may be potential biomarkers related to the pathogenesis of obesity-related T2DM.

## 1. Introduction

Type 2 diabetes mellitus (T2DM) is characterized by insulin resistance and a relative defect in insulin secretion [1, 2]. Obesity, as a low-grade chronic inflammation defined recently, can induce the activation of innate immunity and complicated adaptive immune regulation which play an important role in the onset as well as the progression of T2DM [3]. Moreover, there are a large number of studies that focus on the link between obesity and insulin resistance [4, 5].

T cell immunoglobulin and mucin domain-containing protein 3 (TIM3), which belongs to the T cell immune globulin sticky protein molecule family, was originally reported to be expressed on Th1 cells. By binding to its ligand galectin-9, TIM3 can induce T cell exhaustion and tolerance [6, 7]. Increasing evidence showed the vital role of the interaction of TIM3 and galectin-9 in several chronic diseases, such as cancer, hepatitis, and autoimmune diseases [8, 9]. The physiological role of TIM3 has been implicated in both stimulatory and inhibitory functions [10, 11]. The biological effect of TIM3 has been controversially discussed because of its costimulatory and coinhibitory properties. Patients of autoimmune diseases displayed altered frequencies and function of CD4^+^ T cells, CD8^+^ T cells, and monocytes, as well as a different expression of surface markers. The expression of TIM3 on T cells is downregulated in rheumatoid arthritis and Graves' disease, which revealed its inhibitory properties. In contrast, the number of TIM3 and galectin-9-positive CD4^+^ T cells was increased at the early stages of multiple sclerosis [12]. Yan et al. found that TIM3 on CD14^+^ monocytes might act as a novel biological marker for diabetes duration in type 2 diabetes mellitus [13], which is consistent with the costimulatory properties of this molecule. However, it is unclear whether TIM3 is involved in the pathogenesis of obesity progression to T2DM.

In this study, we examined the expression of TIM3 and galectin-9 in the peripheral blood of patients with obesity and obesity-related T2DM to evaluate its role in the pathogenesis of obesity-related T2DM. We also analyzed the correlation with clinical indicators.

## 2. Materials and Methods

### 2.1. Patients and Controls

The study protocol was approved by the Ethics Committee of First Affiliated Hospital of Soochow University, and written informed consent was obtained from each individual. The study enrolled 25 nonobese, diabetes-free healthy donors (HC); 25 patients with simple morbid obesity (OB); and 25 patients with obesity and T2DM (OD). All the subjects are age- and sex-matched. Clinical characteristics for the patients and controls are presented in Table 1. Using the Working Group on Obesity in China (WGOC) criteria, obesity is defined as a BMI ≥ 28 kg/m^2^. Normal BMI was defined as derived measures between 18.5 and 24 kg/m^2^. The diagnostic criteria for T2DM were according to the World Health Organization (WHO) diagnostic criteria (http://www.who.int/diabetes/publications/diagnosis_diabetes2006/en/). All the patients are newly diagnosed, with no history of medication. Fresh whole blood was collected into tubes containing EDTA and stored on ice. The plasma was immediately centrifuged at 2000 × g for 10 minutes at 4°C and stored at -80°C until the assays were performed. The peripheral blood mononuclear cells were used for flow cytometry detection.

### 2.2. Antibodies and Flow Cytometry

FACS stainings were performed on the peripheral blood mononuclear cells. The following antihuman monoclonal antibodies were used from BioLegend: TIM3-PE (clone: F38-2E2), antihuman CD3-APC (clone: UCHT1), CD4-FITC (clone: RPA-T4), and CD8-FITC (clone: HIT8a). And the respective isotype control immunoglobulin G was involved as the control. For surface stainings, 100 *μ*L peripheral blood mononuclear cells were stained with the appropriate volume of pretitred antibody for 20-30 min at RT, then red blood cells were lysed, and white blood cells were immobilized by using OptiLyse C Lysing Solution. Cells were incubated and evaluated using a COULTER Epics XL flow cytometer (Beckman Coulter), and the gating strategy is described in Supplementary Figure 1. Data were acquired on flow cytometry (Beckman Coulter, CA) and analyzed using the FlowJo software (TreeStar, OR).

### 2.3. Soluble Galectin-9 Enzyme-Linked Immunosorbent Assay (ELISA)

The plasma levels of galectin-9 were measured quantitatively using a solid-phase enzyme-linked immunosorbent assay according to the manufacturer's instructions (Catalog NO: EK1113, BOSTER, China). This assay has a high sensitivity (minimum detectable dose: 10 pg/mL), and no significant cross-reactive or interference was observed. The calibration and standardization of the assay were performed according to the manufacturer's protocol. The plates were read in a microplate reader (Bio-Rad, CA) for the absorbance at 450 nm.

### 2.4. Statistical Analysis

Statistical analysis was performed by GraphPad Prism 5 (San Diego, CA). All the quantitative data were presented as the mean ± standard deviation (SD). The unpaired *t* test or Mann–Whitney *U* test was used for comparison between groups according to whether they conform to the normal distribution. Correlations between continuous variables were analyzed by Spearman's rank correlation test. *P* values less than 0.05 were considered as a significant difference.

## 3. Results

### 3.1. Patient Cohorts

This study population includes 25 healthy controls (HC group), 25 cases of simple obesity (OB group) and 25 cases of obesity comorbid T2DM (OD group). The mean BMI was 21.96 ± 1.99, 31.64 ± 2.30, and 32.25 ± 4.69, respectively. The mean HbA1c value in each group was 5.35 ± 0.35, 5.56 ± 0.35, and 10.38 ± 2.11 mg/dL, respectively. The demographic data for the cohorts is summarized in Table 1.

### 3.2. TIM3 Expression Is Upregulated on CD8-Positive T Lymphocytes in Patients with Obesity

**Figure 1:**
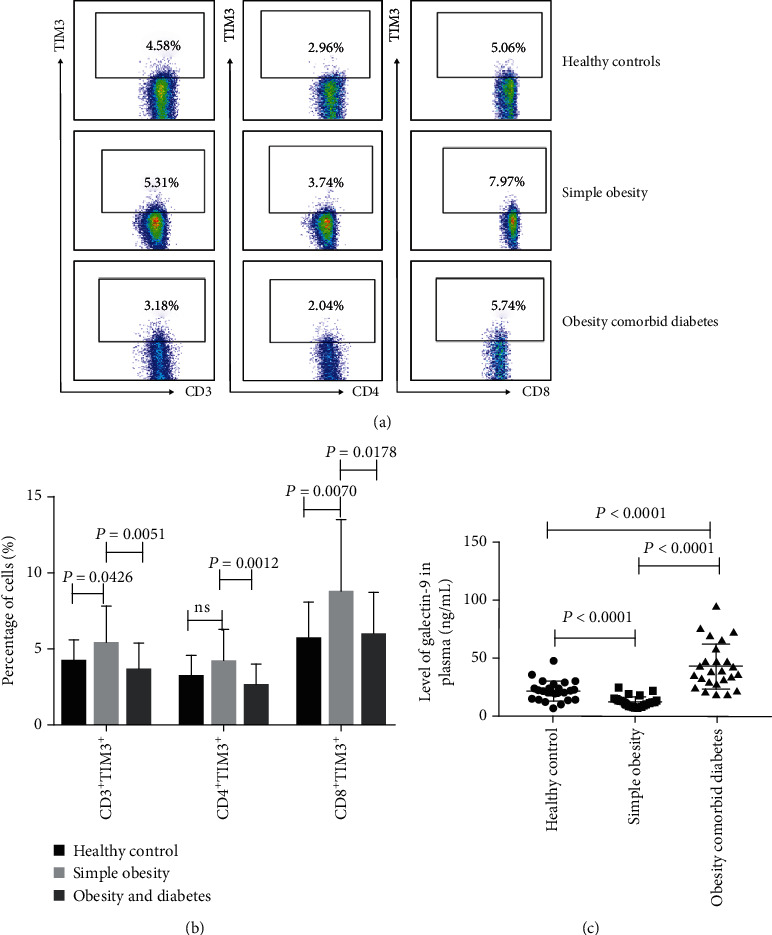
TIM3 and galectin-9 expression in healthy controls, obesity, and obesity-related diabetes patients. (a) The expression level of TIM3 was detected by the flow cytometry of CD3^+^, CD4^+^, and CD8^+^ T cells from the HC, OB, and OD groups. (b) Percentages of CD3^+^TIM3^+^, CD4^+^TIM3^+^, and CD8^+^TIM3^+^ cells in the PBMC samples of patients from the HC, OB, and OD groups. (c) Expression of galectin-9 in the plasma of the HC, OB, and OD groups.

**Figure 2:**
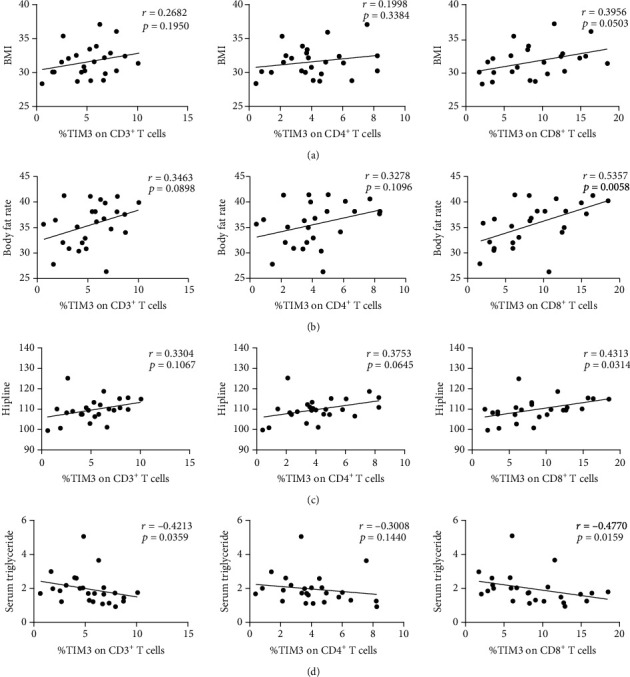
Correlation analyses between TIM3 expression and clinical indicators in obesity patients. Correlation analysis of TIM3-positive cells and BMI (a), body fat rate (b), hipline (c), and triglyceride (d).

Firstly, we investigated TIM3 expression on T cells upon chronic infection in obesity. Our results showed that the expression of TIM3 on CD3^+^, CD4^+^, and CD8^+^ T cells was increased in obese patients compared with HC, respectively (5.38 ± 2.44% vs. 4.19 ± 1.48%, *P* = 0.0426; 4.14 ± 2.11% vs. 3.21 ± 1.35%, *P* = 0.0709; 8.72 ± 4.76% vs. 5.68 ± 2.32%, *P* = 0.0070, respectively) (Figures 1(a) and 1(b)). The correlations between the TIM3 expression and disease activity as well as clinical parameters were then examined. As shown in Table 2 and Figure 2, in the OB group, the TIM3 expression on CD8^+^ T cells was positively correlated with BMI, body fat rate, and hipline (*r* = 0.3956, *P* = 0.0503; *r* = 0.5357", *P* = 0.0058; *r* = 0.4313, *P* = 0.0314, respectively) and negatively correlated with serum triglyceride and creatinine (*r* = −0.4770, *P* = 0.0159; *r* = −0.6354, *P* = 0.0006, respectively). However, the expression of TIM3 either on CD4^+^ or on CD3^+^ T cells was not significantly correlated with these parameters. These results revealed a distinct correlation between the TIM3 expression on CD8^+^ T cells and obesity, which may suggest the vital role of TIM3^+^CD8^+^ T cells in the development of obesity.

### 3.3. Reduced TIM3 Expression in Patients with Obesity Comorbid T2DM

To investigate the role of TIM3 in the progression of obesity to type 2 diabetes, we collected blood specimens from 25 patients with obesity comorbid T2DM. The expression of TIM3 on CD3^+^, CD4^+^, and CD8^+^ T cells in the OD group was significantly lower compared with that in the OB group (3.57 ± 1.80% vs. 5.38 ± 2.44%, *P* = 0.0051; 2.60 ± 1.40% vs. 4.14 ± 2.11%, *P* = 0.0012; 6.00 ± 2.83% vs. 8.72 ± 4.76%, *P* = 0.0178, respectively). Compared with the healthy controls, only the frequencies of CD4^+^TIM3^+^ T cells were lower. There was no significant correlation between the expression of TIM3 and any clinical features such as BMI, hipline, HbA1c, fasting C-peptide, LDL, HDL, and TG of T2DM (Table 3). Maybe the transient high glucose upregulates the TIM3 expression on peripheral T cells during the progression of obesity, while long diabetes duration restores T cell function by downregulating the TIM3 expression.

### 3.4. Galectin-9 Was Decreased in Obesity Patients but Increased in Patients with Obesity and T2DM

**Figure 3:**
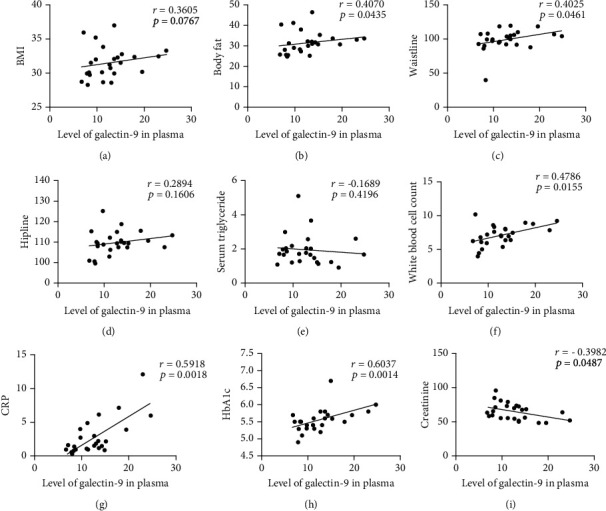
Correlation analyses between the galectin-9 expression and clinical indicators in obesity patients. Correlation analysis of plasma galectin-9 and BMI (a), body fat percentage (b), waistline (c), hipline (d), triglyceride (e), white blood cell count (f), CRP (g), HbA1c (h), and creatinine (i).

The level of galectin-9 in plasma was determined by ELISA. As shown in Figure 1(c), the concentration of galectin-9 in the plasma of the OB group was significantly lower than that of HC and OD group (12.75 ± 4.70 vs. 22.19 ± 8.68, *P* < 0.0001; 12.75 ± 4.70 vs. 43.94 ± 19.5, *P* < 0.0001). Diabetic patients with obesity had higher galectin-9 expression than nondiabetic obese patients. A significant positive correlation was found between galectin-9 level and body fat (*r* = 0.4070, *P* = 0.0435), waistline (*r* = 0.4025, *P* = 0.0461), CRP (*r* = 0.5918, *P* = 0.0018), and HbA1c (*r* = 0.6037, *P* = 0.0014) in obesity patients, but there were no significant correlations between galectin-9 and BMI, LDL, HDL, and TG (Figure 3, Table 2). This result showed that galectin-9 might play a different role in the pathogenesis of obesity and T2DM. Galectin-9 may serve as a potential marker for diabetes initiation.

### 3.5. The Level of Galectin-9 in the Plasma of Obese Comorbid T2DM Patients Correlates Positively with the Indicators of Islet Function

**Figure 4:**
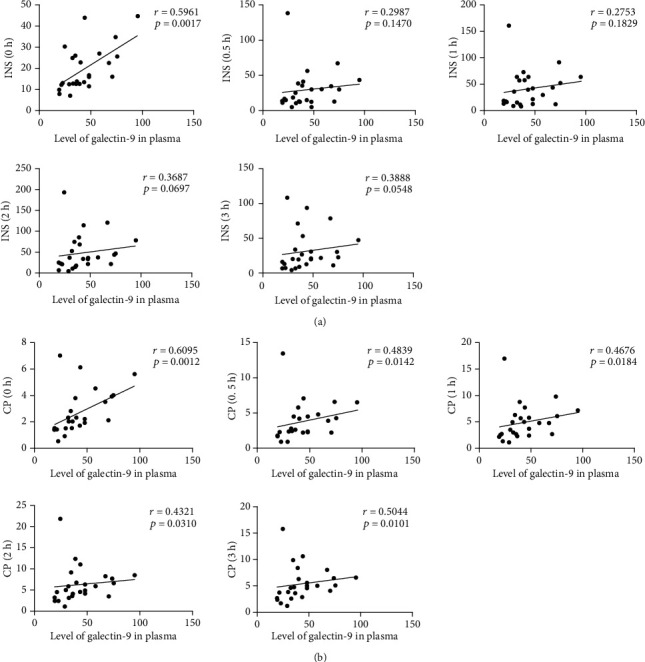
Correlation between galectin-9 level and indicators of islet function in obese T2DM patients. (a) Correlation between galectin-9 level and insulin (INS) at 0 min (0 h), 30 min (0.5 h), 60 min (1 h), 120 min (2 h), and 180 min (3 h). (b) Correlation between galectin-9 level and C-peptide (CP) at 0 min (0 h), 30 min (0.5 h), 60 min (1 h), 120 min (2 h), and 180 min (3 h).

Type 2 diabetes mellitus is characterized by the progression from insulin resistance to insufficient insulin secretion. Both insulin and C-peptide release tests can reveal the function of the islet. A significant positive correlation was found between fast insulin (0 h), C-peptide (0 h), and galectin-9 level (*r* = 0.5961, *P* = 0.0017; *r* = 0.6095, *P* = 0.0012). Other test indexes are almost all related to galectin-9 level including CRP and UA (*r* = 0.5170, *P* = 0.0097; *r* = 0.4021, *P* = 0.0514) (Table 3, Figure 4). The above results indicated that galectin-9 level could be used as a new biological indicator of islet function in obese comorbid T2DM patients, that is, the worse islet function was and the lower galectin-9 level was. Galectin-9 could aid in clinical decision-making in obesity-related T2DM.

## 4. Discussion

Obesity is characterized by a chronic, low-grade inflammation and is shown to be a critical predisposing factor for T2DM development. It can interfere with the balance of the body's immune system by infiltrating the proliferating adipose tissue and releasing inflammatory factors [14]. However, the role of peripheral T cells when in the progression from obesity to diabetes remains unclear.

Increasing studies have shown that TIM3 could inhibit T cell function in autoimmune diseases via suppressing cell-mediated as well as humoral immune responses. TIM3 serves not only as an important regulator of TH1, TH17, NK cells, and monocytes but also as a biomarker of cellular activation [6, 15–17]. It was reported that the TIM3 pathway blockade could accelerate diabetes and promote immunological tolerance in nonobese diabetic mice [18]. In this way, TIM3 may negatively regulate TH1-dependent immune responses and thus affect the progression of autoimmune diseases. On the other hand, a recent literature found that through promoting macrophage activation via the NF-*κ*B pathway, TIM3 could aggravate podocyte injury in diabetic nephropathy [19]. Meanwhile, in type 2 diabetes with chronic kidney disease, Kurose et al. demonstrated that the serum level of galectin-9 was upregulated and closely linked to glomerular filtration rate, which might be related to the alteration of the immune response and inflammation [20]. However, it is still unclear whether the TIM3/galectin-9 signal is involved in the pathogenesis of obesity progression to type 2 diabetes. Therefore, in the present study, we evaluated the expression of TIM3 and galectin-9 in peripheral blood among healthy donors, patients with obesity, and obesity patients with newly diagnosed type 2 diabetes.

We found that the expression of CD3^+^, CD4^+^, and CD8^+^ T cells was increased in obese patients which was consistent with the previous study [21]. CD8^+^ T cells may play a pathogenic role in the early stages of obesity-related inflammation. A recent study showed that TIM3^+^CD8^+^ T cells may sustain the potential for IFN-*γ* production but lose cytotoxic activity in ovarian cancer [22]. Also, insulin resistance could induce the frequency of TIM3 expressing CD8^+^ T cells in breast cancer [23]. In our study, there was a significant increase in the TIM3 expression on CD8^+^ T cells in obese patients compared with HCs, and we found the positive correlation between TIM3 expression on CD8^+^ T cells and BMI, body fat percentage, and hip circumference. All these results may reflect the increased inflammatory status and insulin resistance in obese patients. TIM3^+^CD8^+^ T cells may serve as a novel biological marker for obesity, but the exact function needs to be expounded further.

However, when we compared the TIM3 expression between obese and obese with the T2DM group, it downregulated significantly in obese comorbid T2DM patients, even lower than that of healthy controls. Given the important role of TIM3 in insulin resistance [24], it may reveal that during obese, islet *β*-cells initially upregulated insulin secretion in order to compensate for insulin resistance. Once the patients had developed diabetes, TIM3 became dysfunctional and downregulated. The lower expression of TIM3 may also lose the inhibitory ability to CD4^+^ T cells, which are involved in the pathogenesis of T2DM [25, 26].

Galectin-9 can regulate T cell function in the TIM3 independent way. Chen et al. demonstrated that as soon as T cells were activated, endogenous galectin-9 was recruited to immune synapses and then enhanced TCR signaling to potentiate autoimmune diseases [27]. Overexpression of galectin-9 in islets could prolong grafts survival in NOD/SCID mice, suggesting galectin-9 may be released from pancreatic *β*-cells to terminate TH1-mediated inflammation [28]. Many evidences demonstrated that galactin-9 was a novel, easy to measure biomarker for type 1 IFN signatures [29]. In our study, plasma galectin-9 levels in patients with obesity comorbid type 2 diabetes significantly increased and positively correlated with weight, BMI, hipline, UA, and especially islet-related indicators insulin and C-peptides. These results were different with those from patients with T2DM and chronic kidney disease [8]. It may be due to the inflammatory status of obese-associated insulin resistance, as well as the alteration of the immune response of the patients with obesity comorbid T2DM. Thus, we hypothesized that TIM3 was constitutively expressed on peripheral T cells at a certain level, and as soon as obesity occurred, galectin-9 could be downregulated. While HLA-B-associated transcript 3 (BAT3) was bound to its cytoplasmic tail and recruited the active, catalytic form of tyrosine kinase LCK, T cell activation was permissive by TIM3. But during the progression from obesity to diabetes, galectin-9 could upregulate and trigger phosphorylation of Tyr256 and Tyr263 by the tyrosine kinase ITK, thereby allowing TIM3 to exert the inhibitory function. During its gradual progress to diabetes, in order to maintain the cell in a quiescent state, TIM3 can no longer be regulated.

There are some acknowledged limitations in this study. Firstly, since the sample size of this study is small, the conclusions in our findings need to be confirmed in further studies with a larger size. Secondly, the expression of TIM3 mainly focused on T cells without exploring other types of immune cells. Finally, the distinct mechanism of the TIM3/galectin-9 pathway in the pathogenesis of obesity-related diabetes should be further explored.

In general, the expression of TIM3 on CD3^+^, CD4^+^, and CD8^+^ T cells in patients with obesity was increased, while they went down significantly in patients with obesity and T2DM. The level of galectin-9 in the plasma of obesity patients was significantly lower than that of healthy donors and obesity-related T2DM patients. Moreover, the level of galectin-9 has a markedly positive correlation with both fasting insulin and C-peptide, which represent the function of the islet in T2DM. Our data suggest that the TIM3/galectin-9 pathway might participate in the proceeding from obesity to T2DM, but the exact role of TIM3 in obesity-related T2DM needs to be further elucidated.

## Figures and Tables

**Table 1:** Clinical and biochemical characteristics of the patients.

Characteristics	Healthy control (25)	Simple obesity (25)	Obesity and diabetes (25)
Sex (male/female)	10/15	12/13	13/12
Age (y)	36.76 ± 8.92	33.04 ± 6.41	40.60 ± 14.05
Weight (kg)	60.84 ± 7.37^∗∗∗∗^	89.24 ± 11.3	92.46 ± 17.65
BMI (kg/m^2^)	21.96 ± 1.99^∗∗∗∗^	31.64 ± 2.30	32.25 ± 4.69
Body fat (kg)	—	31.72 ± 5.46	—
Body fat rate (*n*%)	—	35.61 ± 4.28	—
Waistline (cm)	—	99.39 ± 15.38	106.6 ± 13.00
Hipline (cm)	—	109.90 ± 5.69	111.4 ± 11.75
SBP (mmHg)	120.30 ± 12.26	123.80 ± 13.14^#^	132.00 ± 14.19
DBP (mmHg)	76.08 ± 22.18^∗∗∗^	84.68 ± 9.24	86.92 ± 10.53
FBG (mmol/L)	4.56 ± 0.41	4.90 ± 1.33^####^	13.67 ± 21.07
HbA1c (mg/dL)	5.35 ± 0.35	5.56 ± 0.35^####^	10.38 ± 2.11
TC (mmol/L)	4.09 ± 0.56^∗∗∗∗^	5.07 ± 0.72	5.43 ± 2.20
TG (mmol/L)	0.87 ± 0.40^∗∗∗∗^	1.95 ± 0.91^###^	3.32 ± 2.48
HDL (mmol/L)	1.33 ± 0.24^∗∗∗∗^	1.00 ± 0.16^##^	0.85 ± 0.22
LDL (mmol/L)	2.45 ± 0.52^∗∗^	2.97 ± 0.72	2.94 ± 1.00
CRP (mmol/L)	—	2.76 ± 2.74^#^	5.04 ± 4.00
Creatinine (*μ*mmol/L)	56.61 ± 12.57^∗∗∗^	64.72 ± 12.33	51.83 ± 14.85
UA (*μ*mmol/L)	299.03 ± 66.31^∗^	393.30 ± 92.46^##^	416.20 ± 110.60
WBC (10^9^/L)	5.98 ± 1.72^∗^	7.07 ± 1.51	7.23 ± 2.14
Lymphocyte count (10^9^/L)	1.88 ± 0.52	2.20 ± 0.72	2.25 ± 0.58
INS (0 h) (*μ*U/mL)	—	19.32 ± 11.64	19.68 ± 10.44
INS (2 h) (*μ*U/mL)	—	100.80 ± 70.65^####^	48.90 ± 43.45
CP (0 h) (ng/mL)	—	2.74 ± 1.27	2.73 ± 1.67
CP (2 h) (ng/mL)	—	9.12 ± 3.69^##^	6.29 ± 4.26
GC (0 h) (pg/mL)	—	225.30 ± 179.40	194.80 ± 151.70
GC (2 h) (pg/mL)	—	298.50 ± 563.90	193.50 ± 144.60
Diabetic duration (months)	—	—	26.73 ± 42.06
CD3^+^ T cells (%)	67.42 ± 6.12	70.42 ± 4.90	70.85 ± 7.09
CD4^+^ T cells (%)	40.67 ± 7.34	42.16 ± 7.03^#^	46.73 ± 7.12
CD8^+^ T cells (%)	23.43 ± 6.12	24.45 ± 5.64	22.00 ± 6.46

The data are mean ± standard deviation. BMI: body mass index; SBP: systolic blood pressure; DBP: diastolic blood pressure; FBG: fasting blood glucose; HbA1c: glycosylated hemoglobin; CRP: C-reactive protein; TC: serum total cholesterol; TG: serum triglyceride; HDL: high-density lipoprotein; LDL: low-density lipoprotein; UA: uric acid; WBC: white blood cell count; INS: serum insulin; CP: C protein; GC: glucagon. ^∗^Healthy control versus simple obese. ^#^Simple obese versus obese with diabetes. ^∗^*P* < 0.05; ^∗∗^*P* < 0.01; ^∗∗∗^*P* < 0.001; ^∗∗∗∗^*P* < 0.0001; ^#^*P* < 0.05; ^##^*P* < 0.01; ^###^*P* < 0.001; ^####^*P* < 0.0001.

**Table 2:** Correlation between TIM3, galectin-9, and clinical indicators in simple obesity patients.

	CD3^+^TIM3^+^		CD4^+^TIM3^+^		CD8^+^TIM3^+^		Galectin-9	
	*r*	*P*	*r*	*P*	*r*	*P*	*r*	*P*
Weight	0.0868	0.6800	0.0707	0.7371	0.0739	0.7255	0.1143	0.5865
BMI	0.2682	0.1950	0.1998	0.3384	0.3956	0.0503	0.3605	0.0767
Body fat	0.3889	0.0547	0.2015	0.3340	0.5115	0.0090	0.4070	0.0435
Body fat rate	0.3463	0.0898	0.3278	0.1096	0.5357	0.0058	0.1851	0.3757
Waistline	0.2632	0.2037	0.2224	0.2854	0.2185	0.2940	0.4025	0.0461
Hipline	0.3304	0.1067	0.3753	0.0645	0.4313	0.0314	0.2894	0.1606
FBG	-0.1343	0.5220	-0.0289	0.8911	-0.1004	0.6329	-0.01886	0.9287
HbA1c	0.2969	0.1495	0.2672	0.1966	0.2676	0.1959	0.6037	0.0014
TC	0.1209	0.5648	0.1833	0.3805	0.1370	0.5136	0.0046	0.9825
TG	-0.4213	0.0359	-0.3008	0.1440	-0.4770	0.0159	-0.1689	0.4196
HDL	0.1577	0.4516	0.3172	0.1223	0.2212	0.2879	0.199	0.3402
LDL	0.1246	0.5528	0.1091	0.6038	0.1727	0.4090	0.0789	0.7079
CRP	0.3940	0.0513	0.3208	0.1179	0.4066	0.0437	0.5918	0.0018
Creatinine	-0.4811	0.0149	-0.5433	0.0050	-0.6354	0.0006	-0.3982	0.0487
UA	-0.3176	0.1218	-0.3273	0.1102	-0.3697	0.0689	-0.1496	0.4753
White blood cell count	0.5849	0.0021	0.5720	0.0028	0.5957	0.0017	0.4786	0.0155
Lymphocyte count	0.4348	0.0299	0.2713	0.1897	0.5332	0.0061	0.1674	0.4239
INS (0 h)	0.1747	0.4036	0.1239	0.5552	0.1570	0.4537	0.2262	0.2768
INS (2 h)	0.1566	0.4548	0.1262	0.5479	0.1877	0.3690	0.3235	0.1147
CP (0 h)	0.0522	0.8043	-0.0720	0.7323	0.0054	0.9796	-0.1007	0.6319
CP (2 h)	0.0599	0.7760	0.0103	0.9611	0.0961	0.6477	0.3541	0.0825
GC (0 h)	-0.2853	0.2832	-0.1000	0.7132	-0.2559	0.3376	0.1192	0.6583
GC (2 h)	-0.0941	0.7296	0.1265	0.6405	-0.0088	0.9781	0.1692	0.5282

Abbreviations: BMI: body mass index; FBG: fasting blood glucose; CRP: C-reactive protein; TC: serum total cholesterol; TG: serum triglyceride; HDL: high-density lipoprotein; LDL: low-density lipoprotein; UA: uric acid; INS: serum insulin; CP: C protein; GC: glucagon.

**Table 3:** Correlation between TIM3, galectin-9, and clinical indicators in obesity comorbid T2DM patients.

	CD3^+^TIM3^+^		CD4^+^TIM3^+^		CD8^+^TIM3^+^		Galectin-9	
	*r*	*P*	*r*	*P*	*r*	*P*	*r*	*P*
Weight	0.2410	0.2459	0.2298	0.2692	0.2161	0.2995	0.5275	0.0067
BMI	-0.0462	0.8265	-0.1212	0.5639	-0.0796	0.7052	0.5620	0.0035
Waistline	-0.2942	0.1534	-0.2457	0.2366	-0.3769	0.0633	0.3189	0.1203
Hipline	0.0380	0.8569	-0.0976	0.6425	-0.0320	0.8792	0.5322	0.0062
FBG	0.2414	0.2559	0.2427	0.2531	0.1766	0.4092	-0.1227	0.5678
HbA1c	-0.0222	0.9180	-0.0166	0.9388	0.0473	0.8261	0.0026	0.9903
TC	0.1327	0.5366	0.2291	0.2816	0.2756	0.1925	-0.1014	0.6373
TG	0.0896	0.6772	0.2727	0.1973	0.1018	0.6361	-0.1044	0.6272
HDL	-0.2336	0.2719	-0.2343	0.2704	-0.1712	0.4237	-0.3644	0.0800
LDL	0.2766	0.1907	0.1471	0.4929	0.3385	0.1056	0.0527	0.8069
CRP	-0.2100	0.3246	-0.0009	0.9968	-0.1265	0.5557	0.5170	0.0097
Creatinine	0.00057	0.9791	0.0948	0.6594	-0.1410	0.5111	-0.1382	0.5197
UA	0.0074	0.9727	0.1857	0.3849	-0.1658	0.4388	0.4021	0.0514
White blood cell count	-0.0670	0.7505	0.0389	0.8537	0.0121	0.9541	0.1863	0.3725
Lymphocyte count	0.0333	0.8745	0.2482	0.2315	-0.0015	0.9942	0.1371	0.5136
INS (0 h)	0.1270	0.5451	0.3480	0.0883	0.0716	0.7338	0.5961	0.0017
INS (0.5 h)	-0.1705	0.4153	-0.0200	0.9244	-0.2793	0.1764	0.2987	0.1470
INS (1 h)	-0.0535	0.7995	0.0820	0.6969	-0.2157	0.3005	0.2753	0.1829
INS (2 h)	-0.1216	0.5626	0.0377	0.8580	-0.3101	0.1315	0.3687	0.0697
INS (3 h)	-0.1170	0.5777	0.1270	0.5453	-0.2516	0.2251	0.3888	0.0548
CP (0 h)	-0.0295	0.8888	0.1268	0.5459	-0.1347	0.5210	0.6095	0.0012
CP (0.5 h)	-0.1447	0.4900	0.0559	0.7907	-0.2197	0.2914	0.4839	0.0142
CP (1 h)	0.0173	0.9345	0.1773	0.3966	-0.1295	0.5372	0.4676	0.0184
CP (2 h)	-0.1521	0.4680	0.0400	0.8493	-0.3143	0.1260	0.4321	0.0310
CP (3 h)	-0.1443	0.4912	0.0520	0.8052	-0.2653	0.1999	0.5044	0.0101
GC (0 h)	0.1991	0.3401	0.0932	0.6577	0.2246	0.2803	0.0231	0.9127
GC (2 h)	0.2070	0.3319	0.1775	0.4065	0.2006	0.3473	0.0679	0.7525

Abbreviations: BMI: body mass index; FBG: fasting blood glucose; CRP: C-reactive protein; TC: serum total cholesterol; TG: serum triglyceride; HDL: high-density lipoprotein; LDL: low-density lipoprotein; UA: uric acid; INS: serum insulin; CP: C protein; GC: glucagon.

## Data Availability

The data used to support the findings of this study are available from the corresponding authors upon request.

## Abbreviations

T2DM: Type 2 diabetes mellitus
HC: Healthy donors
OB: Patients with simple obesity
OD: Patients with obesity comorbid T2DM
FACS: Flow cytometry.

## Supplementary Materials

Supplementary Materials Figure S1: gating strategy in flow cytometry analysis.
