# Supplementary figures and images for: Circulating T Cells Exhibit Different TIM3/Galectin-9 Expression in Patients with Obesity and Obesity-Related Diabetes
Source: J Diabetes Res. 2020 Oct 15;2020:2583257. doi: 10.1155/2020/2583257 (PMC7585658; doi:10.1155/2020/2583257)

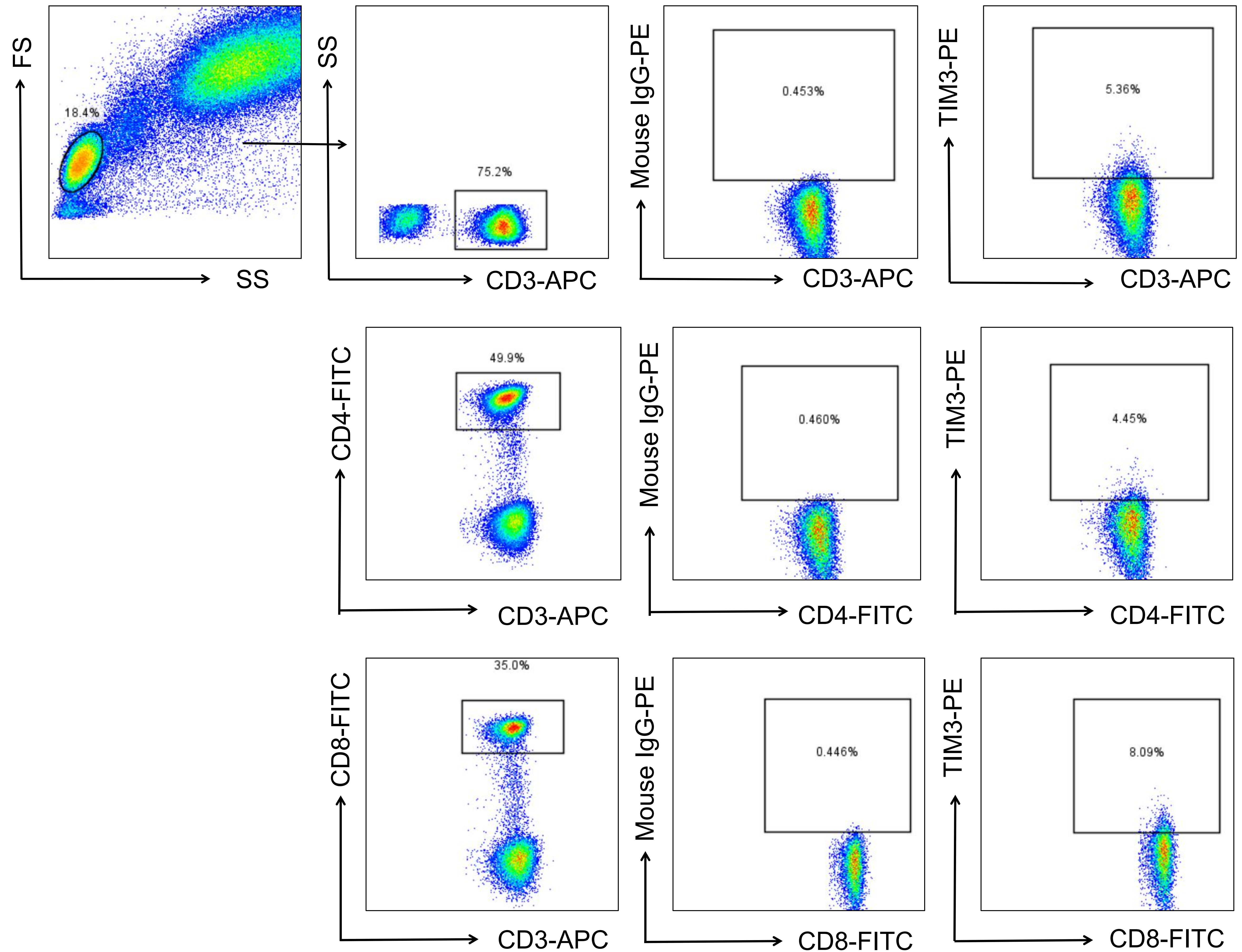

Figure. S1. Gating strategy in flow cytometry analysis.

Supplement: Supplementary Materials — Figure S1: gating strategy in flow cytometry analysis. [file 2583257.f1.pdf]
